# The role of cognitive activity in cognition protection: from Bedside to Bench

**DOI:** 10.1186/s40035-017-0078-4

**Published:** 2017-03-28

**Authors:** Bin-Yin Li, Ying Wang, Hui-dong Tang, Sheng-Di Chen

**Affiliations:** Department of Neurology, Institute of Neurology and the Collaborative Innovation Center for Brain Science, Rui Jin Hospital affiliated to Shanghai Jiao Tong University School of Medicine, Shanghai, 200025 China

## Abstract

**Background:**

Cognitive decline poses a great concern to elderly people and their families. In addition to pharmacological therapies, several varieties of nonpharmacological intervention have been developed. Most training trials proved that a well-organized task is clinically effective in cognition improvement.

**Main body:**

We will first review clinical trials of cognitive training for healthy elders, MCI and AD patients, respectively. Besides, potential neuroprotective and compensatory mechanisms in animal models of AD are discussed. Despite controversy, cognitive training has promising effect on cognitive ability. In animal model of AD, environmental enrichment showed beneficial effect for cognitive ability, as well as neuronal plasticity. Neurotrophin, neurotransmitter and neuromodulator signaling pathway were also involved in the process. Well-designed cognitive activity could benefit cognitive function, and thus life quality of patients and their families.

**Conclusion:**

The positive effects of cognitive activity is closely related with neural plasticity, neurotrophin, neurotransmitter and neuromodulator signaling pathway changes.

## Background

### Cognitive decline and its burden

Cognitive decline is age-specific or related with dementia. Alzheimer disease (AD) was the most common types of dementia. In 2006, the global prevalence of AD was 26.6 million [1]. It is estimated that the one in 85 persons worldwide will suffer from the disease by 2050. In the United States, AD causes estimated health-care costs of $172 billion per year [2]. It was reported that average total costs for AD patients were more than five-fold higher compared with matched controls [3]. If interventions could delay disease onset and slow its progression by a modest 1 year, there would be much fewer cases of the disease in 2050 with reduction by nearly 9.2 million, and thus fewer expenditure on care and treatment [1]. As a continuous course, Alzheimer’s disease (AD) gradually develops from preclinical state, mild cognitive impairment (MCI) to dementia. MCI can thus be regarded as a transitional state between normal aging and AD.

Aging and aging-related diseases pose a major threat to individuals’ life quality, and cause high economic burden on families and the whole society. Prevalence of MCI in population-based epidemiological studies ranges from 3 to 20% in adults older than 60 or 65 years old [4–7]. Some people with MCI seem to remain stable or return to normal over time [5]. However, approximately 50% of patients with MCI (roughly 12% per year) will progress to AD over 4–5 years.

Unfortunately, there is no cure or robust pharmacologic treatment for AD. So far, the primary focus is slowing down the decline of neurological and associated behavioral functions, by providing medications, training and caregiver support.

### Pharmacological and non-pharmacological therapies

Due to the increasing prevalence of MCI and AD, pharmacological and non-pharmacological treatments have been greatly concerned about their effects. Donepezil, rivastigmine, galantamine, and memantine are the drugs presently approved by the Food and Drug Administration (FDA) for treatment of AD. Meta-analysis of cholinesterase inhibitors (ChEIs) showed their effects by a small improvement in activities of daily living [8]. Antiglutamatergic treatment (memantine) reduced clinical deterioration in moderate-to-severe Alzheimer’s disease [9]. In addition, it is still doubted that whether these drugs significantly improve long-term outcomes, such as the need for nursing home admission [10, 11]. Both ChEIs and antiglutamatergic treatment are not indicated for MCI patients. A review and meta-analysis concluded that treatment with cholinesterase inhibitors merely affected MCI progression to dementia or improved cognitive test scores [12]. In one study of data from Alzheimer’s Disease Neuroimaging Initiative, MCI patients who received ChEIs with or without memantine were more impaired, showed greater decline in scores, and progressed to dementia sooner than patients who did not receive ChEIs [13].

As well as medications, non-pharmacological interventions also aim to delay the loss of cognitive abilities, to help people stay independent in everyday life as long as possible, and to increase their well-being and quality of life. Because of its readiness and few side effects, not only AD or MCI patients, healthy elder adults can also participate at home or in the community. There are various approaches, including mental exercise, diet control and physical exercises [14, 15]. However, their effect remains controversial in different clinical trials. Other interventions include art therapy, aromatherapy, music therapy, animal-assisted therapy and caregiver education programs.

#### From bedside to bench

There have been a large number of clinical trials and reviews about the effect of cognitive therapy for aging populations [16–18]. Computerized cognitive training showed its modest effect on cognitive performance in healthy older adults [19], and the effect of cognitive training was equivocal for AD patients [20]. Effective forms of training for AD patients included errorless learning, spaced retrieval, vanishing cues techniques, and the dyadic approach [21]. However, few of these reviews covered clinical outcomes and their underlying mechanisms together.

In this paper, we first review clinical trials of cognitive training for people with different extent of cognitive ability. The characteristics and special outcomes of the clinical trials of cognitive intervention were discussed, as well as shortcomes of clinical studies. Then, much attention was devoted to neural mechanisms of these training, exploring valuable information from animal studies.

## Cognitive activity for human beings

### Method for literature review of clinical trials

We take two steps in the literature review. In step one, we searched relevant meta-analysis for a quick look at the effect of cognitive intervention. In step two, we selected consolidated clinical trials for detailed analysis and discussion.

Step One: Several systemic reviews and meta-analysis have summarized cognitive training trials. Firstly, we selected the work which fully complies with the Preferred Reporting Items for Systematic Reviews and Meta-Analysis (PRISMA) or Delphi list. We searched PubMed- and PsycINFO-based literature for relevant meta-analysis using the following Boolean strategy: “cognitive stimulation” OR “cognitive rehabilitation” OR “cognitive activity” OR “cognitive intervention” OR “training” OR “memory training” OR “mnemonic training”. After screened by title and abstract, meta analysis studies were included if they enrolled latest clinical trials that evaluated the effect of the cognitive intervention by assessing cognitive changes before and after intervention (Table 1).

**Table 1:** Meta-analysis studies for cognitive training in healthy elders, MCI and AD patients

Reference	Participants	Training	Trials	Positive outcomes	Negative outcomes
Lampit, et al., 2014 [19]	Healthy elders	Computerized cognitive training	51	Overall effect; nonverbal, verbal and working memory; processing speed	EF and attention
Toril, P. et al., 2014 [200]	Healthy elders	Vedio game training	20	Reaction time, attention, memory, and global cognition	EF
Kelly, ME et al., 2014 [192]	Healthy elders	Memory-based intervention/diverse stimulations.	31	Executive function/global cognition—compared with active control Memory/subjective cognition—compared with no training group	WM,recall, recognition Recall, attention
Papp, K. et al., 2009 [201]	Healthy elders	Multi-domain training	10	All outcome measures^c^	-
Li, H. et al., 2011 [202]	MCI	Multi-domain training	17	Overall cognition, self-ratings, EM, WM, EF	SM, PS, attention, VS
Martin, M. et al., 2011 [203]	Healthy elders	Multi-domain training	11	Immediate recall^a^	-
			6	Delayed recall^a^	-
			2	-	VS
			5	-	Short-term memory
			1	EF^a^	-
Bahar-F. et al., 2013 [204]	AD + VD	Multi-domain training	11	-	Any reported outcomes
Aquirre, E . et al., 2013 [205]	Dementia ^b^	Cognitive and social function	7	ADAS-Cog	-
Sitzer, DI. et al., 2006 [206]	AD	At least one domain cognitive function	17	Overall cognitive ability	-
Woods, B. et al., 2012 [207]	Dementia ^b^	Cognitive stimulation	7	ADAS-Cog	-

*EF* executive function, *WM* working memory, *MCI* mild cognitive impairment, *EM* episodic memory, *PS* processing speed, *VS* visual-spatial ability, *VD* vascular dementia

^a^Improvements observed did not exceed the improvement in the active control condition

^b^Alzheimer’s disease, vascular dementia mixed Alzheimer’s and vascular dementia, other types of dementia. ADAS-Cog was applied only in AD patients

^c^Significant but negligible

Step Two: In order to further take a close look at the entire literature and details, we manually searched consolidated evidence from the references of selected articles and earlier review [16]. We also searched for published intervention studies in latest 5 years, limiting to English language, human and peer reviewed articles.

The search strategy in PubMed (clinical trials as article type) was shown as an example: (“Dementia”[Mesh] OR “Alzheimer Disease”[Mesh] OR “mild cognitive impairment”[Mesh] OR aging OR elder) AND (cognitive training OR mental training OR train* ) AND (Intervention Studies OR intervention study OR intervention OR interventions OR interventional OR experimental)

We selected studies by three steps based on eligibility criteria of PRISMA checklist : the title and abstract screening, full-text assessment for rationale and eligibility of methodology, and final evaluation of results. Two reviewers (L, BY and W, Y) independently checked the following factors before inclusion, and discrepancy was discussed to arrive at agreement: subjects, study design, blinding, withdrawals and dropouts, intervention method and duration. Studies were excluded if they only enrolled participants with other dementias or no clearly intervention description. Eligible studies were classified by cognition level of subjects, in order to find out training effect on people of different cognitive ability.

### Impact of cognitive activity for healthy older adults

#### Studies description

Normal aging causes natural decline in multiple cognitive domains, and thus poses threat to maintaining independence and quality of life. Researchers tended to develop methods to slow down the speed of cognitive decline, based on the theory that brain retains some plasticity. Since 1980s, a number of studies had tried cognitive interventions for healthy older adults, in order to improve cognitive performance and quality of life [22–25]. These interventions included both laboratory-based and community-based cognitive trainings for healthy older adults.

#### Models of intervention

As memory loss is the major and early complaint from older adults, memory training was dominantly adopted in a number of studies as early as 1980s [26–29]. Working memory training was conducted in 39 80-year-old healthy adults, including visual free-recall tasks [30]. Both training effect (working memory ability) and transfer effect were found improved immediately after training. Nevertheless, after 1-year follow up, both effects were not significant. The pooled data also found its controversial or negligible effect [31, 32]. Spatial working memory task with 2 levels of processing demands helped older adults (70–80 years old) to gain substantial performance on the practiced task. The effects transferred to a more demanding spatial n-back task and to numerical n-back tasks. Both benefits lasted for 3 months, while no evidence was found for far transfer to complex span tasks [33]. Perceptual and verbal working memory training also showed their promising transfer and maintenance effects [34, 35].

More studies paid attention to comprehensive training covering more cognitive domains. Jobe et al. conducted a famous large-scale, randomized, controlled, single-masked trial (ACTIVE study) in 2001, which was designed to determine whether cognitive interventions could affect cognition-based daily functioning and basic trained abilities served as mediators [36]. The ACTIVE study has three models of intervention: speed of processing, memory and reasoning. Each intervention improved the targeted cognitive ability compared with baseline, durable to 2 years. If individuals received booster training 11 months after initial training (2 to 3 weeks), training gains were enhanced and maintained at 2-year follow-up. However, training effect did not transfer to other untrained cognitive skills. In addition, the authors also estimated training effects (cognitive change), compared with expected decline in elderly persons without dementia. The effects from speed of processing, memory and reasoning training were respectively of a magnitude equivalent to the amount of 2-, 7- and 14-year cognitive decline expected in elderly persons without dementia. The study did not find the changes of activities of daily living. The authors attributed it to minimal functional decline across all groups [37]. The study continued and showed improvement of targeted cognitive abilities for 10 years by reasoning and speed, but not memory [38]. Speed of processing training was also believed to be useful in helping driving mobility [39], though its effect was doubted in a new large-sample trials when compared with crossword puzzle game [40].

Donostia Longitudinal Study aimed at all cognitive functions. The authors compared structured training program with unstructured one and control in a 2-year time span. Each participant in experimental groups received 180 ninety-minute sessions (every session per week). Structured intervention covered the cognitive functions of attention and orientation, memory, language, visuoconstructive ability, executive functions, visuomanual coordination and praxia. Only the group that received structured intervention got higher scores in nearly all cognitive tests [41]. Similar computerized cognitive training also boosted memory/attention improvement in the experimental group (word list total score, word list delayed recall, digits backwards, letter-number sequencing) [42], as well as better mood and sleep [43].

In addition to these training programs for particular one or more cognitive domains, social activities or everyday activity were also evaluated as a kind of intervention [44]. Some manipulations included social communication, making sense of figures, drawing activities, and even word-logic puzzles in the community or nursing home [45]. Some training also covered problem-realization and strategy-seeking, helping develop personal strategies for each participant [46]. A large-scale RCT (FINGER study) combined diet, exercise, cognitive training and vascular risk monitoring for improvement or maintenance of cognitive functioning. The results suggested that 2-year multi-domain intervention could improve or maintain cognitive functioning in at-risk elderly people [47].

### Cognitive activity for mild cognitive impairment patients

#### Studies description

Studies varied in the interventions, study design, duration of sessions and sample size. All programs mainly aimed at explicit memory because individuals with MCI suffered from memory deficiency. Similarly to intervention for healthy old adults, attention, speed of processing, language, visual-spatial abilities and executive functions were also adopted [48–51], while others only combined attention and memory training [52, 53]. Computerized cognitive training was also introduced here for some multifaceted interventions [48, 49, 53]. It could facilitate the individual’s approach but did not show increased improvement when compared to non-computerized training, independent of participants’ computer familiarity [54].

#### Interventions for one or more multiple cognitive domains

In the early 2000s, Rapp et al. tried multi-component memory interventions. Patients received education about symptoms of memory loss, memory skills training, and memory-related beliefs. Treated group did better in memory assessment at the end of treatment and at a 6-month follow-up [55].

In a prospective study, Rozzini et al. used training covered different cognitive functions for patients with MCI. After 1-year follow-up, subjects treated with training and cholinesterase inhibitors (ChEIs) medication showed significant improvements in memory, abstract reasoning and in behavioral disturbances. The combined treatment group showed its advantage over ChEIs group [48]. Wenisch et al. administered a cognitive stimulation program (both memory manipulation and information about memory functioning) for cognitive performance. It revealed a larger intervention benefits in MCI than in normal elders’ performances on the associative learning task [56].

In addition to memory, behavior rehabilitation was once believed to be a potential cognitive helper. Nevertheless, it is a bit controversial of its effect in MCI patients. Greenaway et al. tested a calendar/notebook system, including three sections: appointments; daily “to do” items and important events that happened that day. Patients compliant with the system had a medium effect size for improvement in functional ability. Subjects further reported improved independence, self-confidence, and mood [52]. By contrast, Talassi et al. provided a combined cognitive program training and found an improvement in cognitive and affective status of patients with MCI, while no effects were observed in a rehabilitation program not providing a punctual stimulation of cognitive functions [49].

#### Memory strategy intervention

Another critical factor in cognitive training is the use of strategy. Strategy acts as a compensator in functioning. Belleville et al. focused on episodic memory strategies by comparing pre-and post-intervention difference. Progress was remarkable in delayed list recall and face-name association tasks of strategy-intervention group, while no improvement was observed in MCI individuals without receiving the intervention [57]. Hampstead et al. taught MCI patients the use of explicit memory strategies in classic face-name association tasks. Significant improvement was also found on both trained and untrained stimuli, raising the possibility of generalization of training strategies [58].

After being taught with memory strategies based on meta-memory, old adults had training-related gains in a recall task, as well as transfer benefits in short-term memory, long-term memory, working memory and motivation [59]. Strategy-based tasks can be viewed as the acquisition of knowledge that capitalizes on flexibility to improve performance [60]. Performance may increase because the person has acquired knowledge relevant for the particular task.

Londos et al. also aimed at developing compensatory memory strategies that can improve their cognition, as well as occupational performance and quality of life. The study compared cognitive function, occupational performance, and self-perceived quality of life before and after intervention. Significant improvements were seen in cognitive processing speed, occupational performance after participation in the program 2 days per week for 8 weeks [61]. Troyer et al. evaluated the effectiveness of a multidisciplinary intervention, providing evidenced-based memory training and lifestyle education to optimize memory behavior. After treatment, patients had increased memory-strategy knowledge that could change their everyday memory behavior by putting this knowledge into practice. Interestingly, no improvement of objective memory performance was observed [62].

### Cognitive activity for AD patients

#### Studies descriptions

Since publication in the early 1980s by Zarit et al., much work has been done for cognitive stimulation in populations suffering from dementia, especially AD. The early detection and diagnosis of AD raised the importance of effective psychological intervention in the early stage. Types of stimulation programs covered all cognitive domains. Similarly to interventions for normal older adults and MCI patients, memory is the main target, as well as memory strategies and external memory aids. However, due to poor cooperation of AD patients, more delicate techniques were adopted.

#### Memory interventions

In contrary to its general application in MCI patients, memory intervention was questioned when it is applied in AD patients. Quayhagen et al. compared cognitive stimulation with other non-pharmacological interventions (dyadic counseling, dual supportive seminar, and early-stage day care). Cognitive stimulation group demonstrated more improvement over time only in cognitive outcomes [63].

Zarit et al. tried visual imagery for overcoming memory loss in senile dementia patients in community. Though recall performance (imagery techniques were taught) was improved for subjects in the intervention group, the author claimed its little practical value for caregivers [64]. Imaginary memory task (classic face-name association training) was also tried for AD. In a study of 7 AD patients, only one AD patient increased the time during which face-name associations could be held in memory. No training gains were observed for the remaining six patients, thus questioning the generalizability of this method in enhancing memory in dementia [65].

In a combined intervention consisting of face-name associations, spaced retrieval, and cognitive stimulation, 37 patients with probable AD were enrolled for 5 weeks. AD patients who received stimulation showed significant improvement in trained tasks, while no benefit was observed in the additional neuropsychological measures of dementia severity, verbal memory, visual memory, word generation, motor speed, or caregiver-assessed patient quality of life [66].

#### Errorless learning and spaced retrieval

Errorless learning was an instructional design introduced in the 1930s in order to create the most effective learning environment. Errors are once regarded as a function of learning and vice-versa. However, in the errorful learning, people with amnesia much more easily remember their own mistakes than they remember the correction (which is usually the answer they hear from someone else). Errorless learning is an alternative way to get someone to learn something without the opportunity to make a mistake.

In addition to traditional memory or cognitive intervention, errorless learning provides a useful additional strategy. Patients should be tailored to interventions, based on errorless learning principles and specific cognitive problems. In one study, five of the six participants showed significant improvement on the target measures, and maintained this improvement up to 6 months later [67].

Spaced retrieval requires users to rehearse learned information at a certain time. Each new rehearsal is done with a longer or equal interval between itself and the previous rehearsal. At the end of every trial period there is a test phase [68]. Landauer and Bjork first described five types of this learning technique in 1978, and the effectiveness of the rehearsal types was measured by seeing how accurately participants responded during a test phase [69].

Schacter et al. applied the technique to people suffering from amnesia and other memory disorders. Participants were asked to remember some fact with increasing intervals. If the subject is able to recall the information correctly the time is doubled to further help them keep the information fresh in their mind to recall in the future. The findings showed that using spaced retrieval help name face association of young students, as well as individuals with memory disorders [70]. The technique helped demented patients able to place the information in their long-term memory, remembering particular objects names, daily tasks, name face association, information about themselves, and many other facts and behaviors [71]. Spaced retrieval also showed advantage in long-term outcome, which lasted weeks even months later [71].

Spaced-retrieval method was combined with traditional memory stimulation tasks. The term “prospective memory” refers to the timely execution of a previously formed intention. In 1991, it was used for training four AD patients to remember and to implement an intention for future action. All participants were able to shift to new task requirement, and all learned three successive coupon colors successfully [72]. A pilot study designed similar prospective memory tasks, consisting of errorless learning and spaced retrieval techniques. Results showed that AD patients who received prospective memory training performed another similar task successfully across 7 weeks post-treatment [73].

### Training effect on brain activity from clinical perspectives

Neuronal mechanisms underlying the effects of these interventions were investigated by functional magnetic resonance imaging (fMRI) and electroencephalography (EEG). When training and tasks in the fMRI were the same (name-face association) for 6 MCI patients, increased activation was observed in the medial frontal cortex, parietal and occipital lobes neighboring the temporal-parietal junction, left frontal operculum and some areas of the left temporal cortex. It also revealed increased generally connectivity after training, particularly involving the medial temporal gyrus and foci in the occipital and precuneus cortices [74]. In a control study, 2-month verbal memory training improved left hippocampal activation [75], suggesting neuroplasticity related with cognitive training in the hippocampus in MCI.

Cortex also involves in memory trainings, especially when training and tests in fMRI involved different cognitive processes. A combination of specialized areas (activated areas during pre-training and new areas during post-training) was activated in the frontal, temporal and occipital areas. Right inferior parietal lobe was the only activated area that correlated with performance [76]. When tasks were visuospatial mnemonic, occipito-parietal and frontal brain regions had increased activity in younger adults. In older adults, only those that showed increased occipito-parietal activity benefited from the mnemonic. In the next section, more evidence suggested connectivity changes between different brain regions in combined cognitive training.

In gist reasoning training, higher fractional anisotrophy was found after 1-year training using diffusion tensor imaging (DTI) MRI [77]. DTI also revealed microstructural changes of limbic system structures (hippocampus and para-hippocampus) among young adults after a 2-h spatial learning and memory task [78]. A review of 20 research articles indicated that the most robust evidence among elderly adults was a change in anterior hippocampal volume with cognitive activities [79].

Connectivity between different brain areas may change during and after some specific cognitive manipulations. A 6-week training program for healthy older adults on Brain Fitness (an adaptive auditory perception computer game) showed improvement in the activities of daily living (a transfer effect from sensory processing to everyday problem solving). It also selectively increased the integrity of occipito-temporal white matter in the ventral attention network, and decreased connectivity between superior parietal cortex and inferior temporal lobe [80]. This indicates that top-down sensory processing training is associated with improvements in untrained everyday problem solving, depending on changes in the ventral attention network, rather than on the connectivity between the parietal cortex and the temporal lobe.

Neurons interaction could also be evaluated by EEG performance, taking its advantage of high temporal resolution. EEG coherence indicates synchronization between different cortical areas [81, 82]. After training of attention maintenance for 1 month, healthy elder participants demonstrated better performance, with remarkable increase in theta power and long-rang theta coherence between frontal and posterior brain regions [83]. EEG was used to evaluate participants before and after training in one study and revealed neural evidence of functional plasticity in older adult brains. The training-induced modifications in early visual processing during stimulus encoding predicted working memory accuracy improvements [34]. However, cognitive process could not be evaluated by EEG, since it is usually done when patients are under resting state.

Event-related potentials (ERP) [84] were used to find task-related neural discharges. An ERP study for visual search task showed that 10-week training improved attention resource allocation and capacity, by increasing N2pc and P3b amplitudes [85].

Despite the methodological limitations in both fMRI and EEG studies, such as small sample sizes and lack of control groups, these evidence suggested that elder individuals exhibit high plasticity, which can be used as a clue to understand the effects of cognitive interventions.

## Why we turn to bench for more help?

Trials above provided exciting results for clinical practice. However, there are several problems confusing and unsolved. We raise the following reasons that make us turn to laboratory for more help.

At first, the selection and publication bias of clinical trials could not be ignored. AD has its owe pathological characteristics, and preclinical AD happened without any symptoms. Most of the clinical trials only lasted for a relatively short period and only evaluated participants by neuropsychological assessment. It is not sure that they enrolled participants of the correct diagnosis. Besides, some “normal aging” or MCI participants at baseline might progress to AD during follow-up, but few trial corrected the bias when enrollment. In this way, classic animal models of AD may help to find more consolidated evidence for cognitive intervention.

We once reviewed and concluded several principles for an effective training program [86], based on current clinical trials. However, before we design a training program, it is more important to find subjects who really have the indication. One of the most controversial issues of cognitive intervention is its various effect on patients with different level of cognitive ability. Some large-scale studies, such as ACTIVE study, did not precisely differentiate their participants. Positive effect was not proved to retain in participants with or without risk of AD. Though less side effect, cognitive intervention still has costs and is time-consuming. The duration of interventions varies among all studies. In some studies for normal older adults, participants attended the training for as long as 2 years, with a total of 270 h. In more studies, the training period ranged from 3 h to more than 100 h. Every session varied from 30 to 90 min in different studies. What is the relationship between training effect and the duration of intervention? Unfortunately, no one observed the quantitative interaction between duration and extent of improvement. Regarding these uncertainty, it is necessary to figure out who do need and could benefit from such interventions. A retrospective study suggested that neuropsychological profile helped differentiate subjects who respond better [87]. More neurobiological markers might make the intervention a more precise therapy.

Another problem is the duration of effect since intervention ends. The benefits of name-face association training were observed after 1 week [88], or after 1 month [89], and the participants could recognize the training material faster and with fewer errors [58]. Moreover, benefits of speed processing training were observed after 2 years with booster sessions over the course of 11 months [90]. Memory strategy worked as an enhancer of effect sustainment [55, 91], and it was not maintained after three [62] and four [91] months. Goal-oriented cognitive rehabilitation brought increase in self-performance and quality of life, which remained for 6 months [61]. However, the potential mechanisms of the prolonged effect are still unclear. The clarification of neurobiological changes stimulated by cognitive intervention might help a better design of cognitive program.

Finally, though functional MRI provided some evidence for brain activity changes, it has own methodological limitations. Blood oxygenation level dependent contrast (BOLD) in resting or task-related fMRI were mainly used to reflect brain activity. Various statistical methods were adopt to analyze BOLD data. Regional homogeneity, amplitude of low-frequency fluctuation (ALFF), fractional ALFF (fALFF) were once used as representation of regional activities in AD [92, 93], while some used Independent Component Analysis and Granger Causality Analysis to evaluate connectivity [94, 95]. Recently, fMRI validity was seriously questioned for its high false-positive rate from generally used software packages (SPM, FSL, AFNI) [96].

## Cognitive stimulation for animals

Studies above have shown that the progression of dementia could be slowed down in the patients who have cognitively stimulating activities. These discoveries point to the conclusion that cognitive interventions intellectually may not only improve memory performance but also prevent future cognitive decline. Some difficulties met in clinical trials above asked for further exploration in neurobiological way.

### Early experimental studies of brain stimulation on laboratory rodents

We now manipulate mental exercise as stimulating brain which acts a positive role on AD and other forms of dementias via neuroprotective and compensatory mechanism. Existing animal models that are most relevant to our understanding of non-pharmacological therapy include those which utilize environmental or cognitive stimulation as experimental paradigms to alter levels of cognitive activity. In order to attempt to investigate the mechanistic underpinnings of mental exercise in cognitive function, we should first understand early experimental studies of brain stimulation- environmental enrichment (EE) on animal models. EE has defined broadly as the use of housing conditions that offer enhanced sensory, motor, and cognitive stimulation of brain in comparison with standard caging [97]. In the late 1940s, Donald O. Hebb [98] was the first to propose the “enriched environment” as an experimental concept and reported anecdotally that the laboratory rats that he took home as pets solved test problems more easily than the rats kept at the laboratory. While his research did not investigate the brain nor use standardized and enriched environments. More quantitative and controlled EE studies needed to be conducted to test this paradigm systematically. In 1960, Mark Rosenzweig found the rats growing up in the cages with toys, ladders and tunnels showed higher enzyme cholinesterase activity [99]. The following work reported that living in enriched environment altered the function and structure of the brain, and increased cerebral cortex volume [100], thickness [101] and wet weight [102], greater synapse and glial numbers [103]. At that time brain weight and structure were considered a stable characteristic not subject to environmental influences.

From these early conclusions regarding the effects of EE, increasingly refined studies have progressed to showing effects at the cell and molecular levels. Adult rats were placed into enriched housing conditions for 1 year and showed significantly higher levels of nerve growth factor [104]. Another study reported the increased NGF, BDNF and NT-3 protein levels of EE adult rats compared with age-matched isolated condition ones [105]. Researchers also found EE affected the expression levels of a number of genes (microfibrilar protein, microtubule- associated protein 4, PSD-95/SAP90A, Bcl2/Bax, synaptobrevin, for example) involved in neuronal structure, synaptic signaling, and plasticity [106]. As research deepened, investigators have found that EE facilitated repair to the brain in a variety of situations, including severe traumatic brain injury [107, 108], developmental lead exposure [109], and stroke [110] prenatal stress [111], dark rearing [112], and even aging [113].

Numerous cognitive studies about how EE affected the brain had been also proposed. In the intact animals, EE could dramatically improve cognitive abilities [113–115]. In the brain-lesioned animals, EE was beneficial in attenuating cognitive deficits caused by cerebral contusion [107, 110, 116, 117]. Since then, a large volume of literature has evolved describing the effects of EE in a number of different transgenic mouse models of AD (Table 2).

**Table 2:** Cognitive activity effects of enriched environment in animal models

References	Animal models	Age (weeks)	EE Duration (weeks)	Behavior effects	Morphological effects	Molecular effects
Kempermannet et al., 2002 [149]	wild type, C57BL	40	40	Behavioral performance↑	Hippocampal neurogenesis↑	
Veyrac et al., 2008 [157]	wild type, C57BL	8 - 12	7	Short-term olfactory memory ↑	Neurogenesis↑	Noradrenalin levels↑
Frick KM et al., 2003 [113]	wild type, C57BL	12, 104- 108	3	spatial learning task in water maze task ↑		synaptophysin levels↑
Polito L et al., 2014 [128]	APP23, C57BL	12	20, 60	behavioral performance in Water Maze and visual novel Object Recognition Test ↑	Aβ 40/42, pGlu-Aβ 3-40/3-42, or Aβ oligomer level→	BDNF expression↑, sirtuin mRNA and protein levels→
Jeong et al., 2011 [126]	APP, C57BL	12	12 or 24	Cognitive performance↑	Hippocampal neurogensis↑, P-tau at AT8 and AT180 sites ↓, Aβ plaque and levels ↓	
Valero et al., 2011 [154]	APP, C57BL	12	7	Learning and memory↑	neurogenesis ↑, the number of DCX-positive cells↑	
Wolf et al., 2006 [151]	APP23, C57BL	10	34	Water maze performance↑	Hippocampal neurogensis↑, Aβ plaque ↓	NT-3, BDNF levels↑
Costa et al., 2007 [152]	PDGF-hAPP, C57BL	3	18-22	performance of multiple behavioral tasks and memory ↑	Total Aβ and amyloid plaque levels↓	Hippocampal gene expression changed
Jankowsky JL et al.,2005 [119]	APP/PS1, C57BL	8	24	Cognitive function↑	Hippocampal Aβ levels ↑	
Berardi et al., 2007 [123]	AD11, C57BL	8	20	Spatial and visual recognition memory ↑	Aβ burdens↓	Cholinergic deficits↓
Dong et al., 2007 [153]	PS1/2 CKO, B6CBA	4	20	memory performance↑	less enlargement of the lateral ventricles ↑	inflammation-related genes↓
Varman et al., 2013 [174]	Mus booduga	12	4	anxiety-like behavior↓	miR-183 expression↑	acetylcholinesterase level in amygdala of mice ↓
Durairaj et al., 2014 [175]	Mus booduga	12	4	anxiety-like behavioral↓	Hippocampal neurogenesis↑	Dicer, Ago-2 and microRNA-124a expression↑

*Aβ* amyloid beta protein, *P-tau* phosphorylated tau, *pGlu-Aβ 3-40/3-42* Human pGlu-amyloidβ_3–40_ and human pGlu-amyloidβ_3–42_, *AT8* specifically recognizing phospho Ser202/Thr205 tau, *AT180* monoclonal raised against residue Thr231 of PHF-tau, *NT-3* Neurotrophin-3, *BDNF* Brain-derived neurotrophic factor, *Dicer* argonaute RISC catalytic component 2, *Ago-2* argonaute RISC catalytic component 2

### Environmental enrichment-cognitive stimulation on animal models of AD

Many studies had shown that placing animals in complex environments for extended period improved their cognitive performance and brain activity in normal mice and rats. Whether did EE show beneficial effects on behavior and cognition in an animal model of AD? There were several mouse models of neurodegeneration like AD studying the modulating effects of environmental factors: transgenic mice overexpressing amyloid precursor protein (APP) and/or presenilin (PS)-1, AD11 mice expressing anti-nerve growth factor (NGF) antibodies and double transgenic TgCRND8 mice overexpressing the Swedish and Indiana mutations in the human APP. In these researches, the mouse models were given extensive enrichment such as cognitive stimulation or complex housing condition. Cognitive impairment and neuronal alterations elicited by neurodegenerative pathologies were evaluated to determine if they had been ameliorated or rescued by EE. If a long-lasting exposure to EE, the mouse model should display a delayed onset or progression of cognitive impairment.

Before the onset of amyloid formation, APP/PS1 transgenic mice exposed to the long term of EE from 2 to 8 months of age would show mitigated learning and memory deficits. For example, long term EE led to improvement in cognitive function but without decreasing brain beta-amyloid deposition in the aged APPsw mice [118]. Jankowsky, J. L reported that 2-month-old APP/PS1 mice were placed into enriched environment for 6 months, and they swam shorter distances to reach the hidden platform in water maze and more efficiently remembered the platform position. The performance of learning and memory were both normalized to the level of standard-housed non-transgenic mice [119]. Lazarov et al. found pronounced reduction in the levels of cerebral beta-amyloid peptides and amyloid deposits in the same EE APP/PS1 mice [120].

AD11 mice developed age-dependent neurodegeneration including hallmarks of human AD and exhibited progressive memory impairment [121, 122]. Exposed to EE before the onset of behavior deficits for a long time in AD11 mice resulted in preserved visual recognition memory and spatial memory in comparison to non-EE AD11 mice. EE AD11 mice displayed a stronger curiosity when faced a novel object than a familiar object and showed the same ability with wild-type mice on a water maze task [123].

In the TgCRND8 mice, EE had increased exploratory behavior and decreased anxiety-related behavior but could not clearly ameliorate deficits in learning and memory performance [124]. More recent evidence also suggested EE may reduce the cerebral oxidative stress [125], compensate for the effects of stress on disease progression [126], prevent astroglial pathological changes [127] and lessen the cognitive decline [128]. In the Tau-Tg transgenic mice, the NFTs decreased in EE mice [129]. In senescence-accelerated prone mice (SAMP8),EE gave rise to significant beneficial effects at the molecular, cellular, and behavioral levels during brain development, particularly in the hippocampus [130]. In this part, EE provided animals with more novel and complex environment, and thus stimulated cognitive processes, particularly learning and memory. This evidence in AD mice indicated that enhanced cognitive stimulation of EE played a pivotal role in the protection from cognitive impairment.

## What are the mechanisms for the effect of cognitive activity?

### Neuronal circuits

#### Adult neurogenesis

Adult neurogenesis is shown to continue in two parts of brains: the subventricular zone (SVZ) lining the lateral ventricles and subgranular zone (SGZ). In fully adult mammals, new neurons born in SVZ migrate anteriorly into the olfactory bulb (OB), where they mature into local interneurons [131–133]. Adult-generated olfactory interneurons contribute to odor discrimination and olfactory memory [134–136]. It has long been convinced that the hippocampus plays critical role in learning and memory [137], so the production of neurons in the adult hippocampal dentate gyrus (DG) has introduced the possibility of a new form of plasticity that could sustain memory processes. A growing body of evidence support that hippocampal neurogenesis improves pattern separation and spatial memory [138, 139].

Hippocampal neurogenesis could be influenced by several environmental factors and stimuli [140, 141]. While aging was the greatest environmental risk factor, increasing evidence showed noteworthy alteration in neurogenesis took place much earlier than the onset of hallmark lesions or neuronal loss in AD [142, 143]. In aged and AD brains, the proliferation of progenitor cells and their numbers were significantly declined (for review see [144]). The levels of stem cell factor (SCF) which supported neurogenesis in the brain were reduced in the plasma and cerebrospinal fluid of AD patients [145]. In WT mice, EE could enhance hippocampal cell proliferation [146, 147]. And transient receptor potential-canonical 1(TRPC1) was indispensable for the EE-induced hippocampal neurogenesis [148].

Previous studies in transgenic models of AD had generated mounting evidence supporting alterations in neurogenesis. Short-term exposure to EE led to a striking increase in new neurons and a substantial improvement in behavior performance [149]. Studies in several transgenic mice expressing AD-linked gene suggested that adult neurogenesis could be altered by external neurogenic stimulus- enriched environment. EE was reported to increase hippocampal DG neurogenesis and improve their water maze performance in APP23 mice [150, 151]. Enriched housing environment could also improve cognitive performance in PS1/PDAPP transgenic mouse models [152]. In PS1 and PS2 conditional double knockout mice, EE had been shown to be able to induce neurogenesis and effectively enhance memory of the brain [153]. In APP/PS1 double transgenic mice, EE for 7 weeks efficiently ameliorated early hippocampal- dependent spatial learning and memory deficits [154]. Complex environment had been reported to rescue impaired neurogenesis, reduce Aβ levels and amyloid deposition, and significantly enhance hippocampal LTP in APP/PS1 mice [120, 155]. EE applied to SAMP8 at young ages resulted in an increase in NeuN and Ki67 expression [130]. Thus, the proliferation of new neurons which had a reciprocal connection with AD pathogenesis would provide new opportunities for cell therapy for AD.

Although there are lots of studies reporting that EE could increase neurogenesis in DG of the adult hippocampus, neurogenesis in other parts of the brain- the subventricular zone(SVZ) or olfactory bulb (OB) system may be affected by other forms of enhanced stimulation significantly [156]. Olfactory enrichment- a specific form of enhanced sensory stimulation, does appear to increase neurogenesis in the OB [157] and additionally the piriform cortex [158].

#### Neuronal and glial developments

Neurons, neuroglia (including astrocytes, oligodendrocytes and microglia) and ependymal cells make up the complex structure of the adult central nervous system (CNS). Adult neurogenesis bridges between neuronal and glial neurobiology in an intriguing way. When the enrichment environment altered the neurogenesis, the differentiation and development of neuron and glial were always changed at the same time. Increased neuronal differentiation in DG was observed in the EE treated rats and the density of NeuN positive cells was enhanced without new neurons [159]. As many as 90% of cells in the brain are thought to be glial, therefore it is not difficult to understand that the beneficial effects of EE involve glial cell types. There is in fact evidence that EE could alter the numbers of glial in specific brain regions. EE could lead to a significant increase in the number of new astrocytes in layer 1 of the motor cortex [160]. In CA1 region, environmental condition increased the number of astrocytes [161] and stimulated astrocytes to acquire a more stellate morphology [162]. Two months old rats enriched for 7 weeks showed increased antigen expression of both astrocytes and microglia within DG [163]. Glia cells are known to interact extensively with neuron in the brain. Astrocytes secrete factors that promote neuron survival and provide crucial support to neurons. Oligodendrocytes are essential regulators of neurotransmission along myelinated axons. These reports of neuronal effects or non-neuronal effects of EE were interesting in light of evidence that EE plays an important role in modulating neurogenesis and cognition in AD.

### Molecular mechanisms improving cognitive activity

We have now outlined various important structural and cellular changes that have been observed to occur in the animal brain exposure to EE. Evidence in support of such behavioral and cellular effects on molecular mechanisms has been gathered using a range of approaches. Examples of such molecular classes including specific neurotrophins, neurotransmitters and neuromodulator receptors, and synaptic signaling pathways have been validated via gene/protein studies.

#### Activity dependent modulation of gene expression

One early study demonstrated the attenuated expression of AP-2 in the CA2 and CA3 subfield of hippocampus after exposure to EE for 30 days [164]. In adult rats, EE has been shown to upregulate 3H-AMPA binding in the hippocampus by decreasing the capacity of calcium or phosphatidylserine without changes in mRNAs for AMPA receptors [165]. Male rats exposed to EE for 30 days could result in significant higher expression of 5-HT1A receptor mRNA in the hippocampus [166], decreased level of EAAC1 mRNA and increased level of NMDA mRNA specifically in the hippocampus [167]. EE could also increase the mRNA expression levels of 5α-reductase-1 and 3α-hydroxysteroid dehydrogenase, which catalyze synthesis of allopregnanolone from progesterone [168].

A more detailed research analyzed gene expression changes in the cortex of mice and found a large number of genes changed in response to enrichment [106]. Another study analyzed the effects of enriched surroundings with DNA microarrays and found the hippocampus was more responsive to environment stimuli than sensorimotor cortex [169]. Others have also shown the expression of immediate-early gene (IEG) Zif268 could be induced to higher level in the CA3/CA4 region which was associated with enhanced spatial learning task [170]. In NMDAR1-Knockout mice which showed memory impairment, the expression levels of 104 genes involved in multiple signal pathways could be recovered or reversed by EE [171]. Similarly, levels of CREB were increased following EE [172]. With the growing knowledge regarding environment and gene interactions, the framework has been built by an association between gene-environment interactions and disease [173].

Furthermore, there are still numbers of evidence that non-coding RNA species such as microRNAs (MiR) could also be modulated by EE. For example, MiR-183 expression could be upregulated by EE and reduce anxiety-like behavior in mice [174]. MiR-124a showed a similar performance following enriched environment condition [175]. MiR-325 was downregulated in 3 × Tg AD mice but upregulated by EE, which may open new avenues for the studies of treating AD [176]. Until now, this field of exploration is now relatively new, therefore many questions regarding the epigenetic impacts of EE remain unsolved. While the genomic and biochemical tools available are evolving rapidly, there will no doubt be great progress in the near future.

#### Oxidative stress

Previous studies have demonstrated that oxidative stress is an important factor contributing to the onset and progression of AD and the brain is sensitive to oxidative imbalance. Oxidative stress results from increased production of reactive oxygen species (ROS) and reactive nitrogen species (RNS) [177] and possibly precedes Aβ and tau aggregation. The exact mechanisms by how EE provides protection against oxidative damage in the brain with AD remain speculative. In aged rats, complex EE modifies exploration activity, cognition and biochemical markers which may be mediated by oxidative stress levels [178]. Long term exposure to EE from adult age would increase life span in mice [179]. EE rats showed higher values for antioxidant measures and lower values for oxidative stress parameters than control animals [180]. In transgenic mice with Alzheimer-like pathology, cognitive stimulation in the form of EE attenuated pro-oxidative processes and triggered anti-oxidative defense mechanisms by diminishing reactive oxygen and nitrogen species, downregulating pro-inflammatory and pro-oxidative mediators, decreasing expression of pro-apoptotic caspases, and increasing the activities of SOD1 and SOD2 [125]. In another study, EE increased anti-oxidative SOD1 protein and decreased the levels of nitro-tyrosine- a prominent biomarker for oxidative damage [181] .

#### Neurotrophin, neurotransmitter and neuromodulator signaling pathway

Modulation of neurotrophin expression and changes in neurotransmitters and neuromodulators are related to EE according to extensive findings. The primary effect of increased cognitive activity must be via enhanced synaptic and neuronal activity in the relevant neural circuitry. So, it is not difficult to image that molecular effects induced by EE have been shown to involve changes in neurotransmitters. Numbers of studies have shown that EE in animals increased the expression levels of brain-derived neurotrophic factor (BDNF), nerve growth factor (NGF) and vascular endothelial growth factor (VEGF) [182].

In rats with cognitive impairment, the levels of BDNF decreased in the hippocampus and EE exposure could up-regulate the decreased protein levels of BDNF [183]. More studies explained that the enhancement of learning and memory observed after treatment of EE is causally dependent on increased neurogenesis in DG. And BDNF was required for neurogenesis in the adult hippocampus [184] and might be responsible for learning and memory enhancement [185]. EE could significantly increase hippocampal BDNF levels accompanied by increased astrocytes (GFAP+) and microglia (Iba1+) antigen expression [163]. As BDNF supports hippocampal long-term potentiation (LTP), EE also improved synaptic plasticity and cognition through increased levels of BDNF [186].

The experience of APP/PS1 mice in EE would upregulate critical signaling that plays a major role in learning and memory, such as BDNF, IGF-1, N-methyl-D-aspartic acid receptor (NMDAR) and CREB transcripts [187]. NGF is another intensively investigated neurotrophin, when exposed to EE, the levels of NGF was increased either [188, 189] in the cerebral cortex, hippocampal formation, basal forebrain, and hindbrain in EE mice [105]. The levels of NGF mRNA were significant higher in rats housed in a stimulus-rich environment than those in single cages [190]. Short-term EE also increased NGF concentration and improved memory, early neuronal survival in DG [191]. Researchers draw attention to BDNF and NGF mostly because they appeared to be most labile in their expression dynamics and they are important in brain development, function and disease.

## Conclusion and perspectives for the future

As one of non-pharmacological therapies, cognitive activity could benefit cognitive function, and thus life quality of patients and their families. These reviewed clinical studies above highlighted the positive effects of cognitive activity on cognitive ability, well-beings, behavior, and mood in older adults or patients with cognitive decline. We also reviewed the molecular and cellular mechanisms underlying this non-pharmacological therapy.

Based on these clinical trials and meta analysis, we recommend some key features of training that often associated with positive outcomes. Firstly, multi-domain tasks contribute more to cognition [19, 192]. As trained effect could hardly transfer to untrained domains, multi-domain trainings are generally adopted and recommended. Evidence from neuroimaging and animal research suggested that transfer effect could be maximized when tasks are designed to stimulate common brain regions (e.g., hippocampus, striatum) [193, 194]. Besides, challenging tasks for individuals are much more helpful in promoting cognitive ability: training is not learning until the participant can complete the task perfectly. During this period, challenging tasks are helpful for the survival of new neurons [193]. Computerized cognitive training is a lucrative and expanding business. It could easily offer standard and self-adaptive tasks with various levels of difficulty. At the end of training, performance could be evaluated and participants know how well they did. It brings more convenience, as well as less supervision. The researchers identified small but significant effect, while “do-it-yourself” training at home did not produce cognitive improvements [19]. Motivational strategies also can be applied to increase treatment adherence. As described above, training package is recommended, which composes cognitive training package, behavioral therapy for hopelessness and low expectations of success, and a motivational milieu [195]. Compared with control group, AD patients receiving the training package reported fewer depressive symptoms. Fewer depression also leads to better memory improvement and better quality of life [196, 197]. Errorless learning has its special advantage in mild to moderate AD patients. It provides more clues and leads to the only correct answer, which lessens confusion from the difference between correct answer and incorrect response. More importantly, this training process encourages elders to learn by clues in daily life [89, 198].

However, the heterogeneity of cognitive intervention poses great difficulty for a safe conclusion. Combining data from trials of different sample size can result in overestimating the precision of smaller studies. Because of limitation in clinical trials mentioned in Section Why we turn to bench for more help?, we turn to animal studies for more help.

**Fig. 1:**
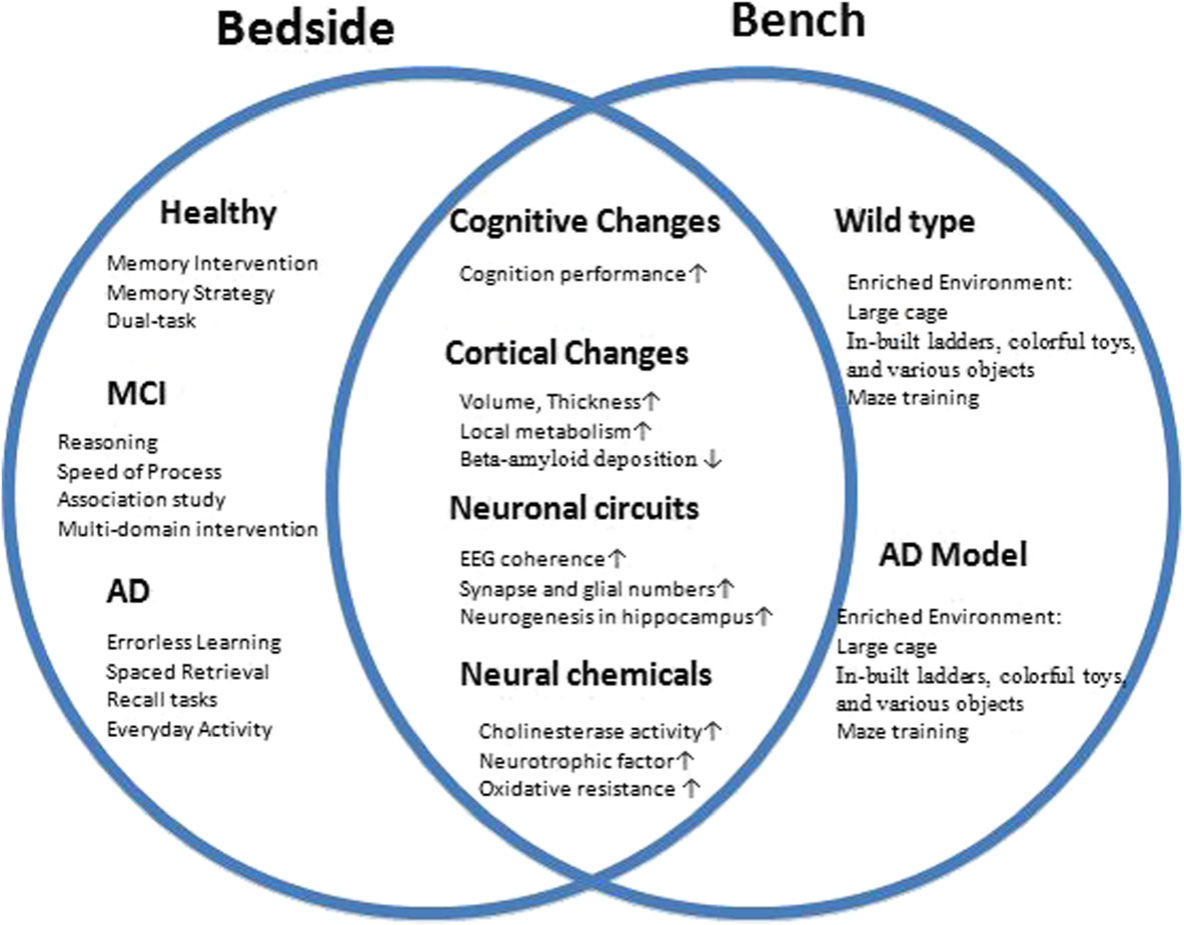
The two circles illustrate the beneficial effect of cognitive training in both clinical and laboratorial studies. The “bedside” semi-circle includes major cognitive trainings that have been tried in healthy old adults, MCI and AD patients. In contrary to heterogeneity of human, mouse models in “bench” semi-circle are nearly monotonous: enriched environment, which offers colorful housing condition including social, sensory and cognitive enrichment. Social enrichment allows more mice dwelling at a large cage to gain companionship and communication. Sensory enrichment provides animals with more novel and complex environments, ladders, colorful toys and various objects for example. And cognitive enrichment stimulates animals’ cognitive processes, particularly learning and memory, in form of maze solving. Exposure to EE could improve animals’ cognitive performance and rescue brain atrophy, which elicited by a number of key molecular and cellular factors, acting at a single neuron or neural circuit level. The shared part of two circles demonstrates neurological effect of interventions for both human and mice, including behavioral, brain structural, neuronal and neural chemicals changes

Evidence from epidemiology and animal model studies suggests that the onset of neurodegenerative diseases could be modulated by environmental factors [199]. However, understanding the mechanism of EE requires animal models showing both behavioral and intrinsic changes which could link data from molecular through to systems levels. The studies compared the behavioral, cellular and molecular data on animal models under EE versus standard conditions. The positive effects of EE include increased adult neurogenesis, elevated or declined gene expression, reduced oxidative stress and subsequently reduced anxiety-like behaviors and improved cognitive performance. This enhanced understanding of EE may provide insight into the mechanistic basis and lead to novel therapeutic approaches which boost endogenous cognitive activity, and thus delay onset of a range of devastating AD and other dementias (Fig. 1).

We find EE has positive influence on normal aging and transgenic AD mouse, which could induce brain structure to produce a variety of changes. The change on anatomy includes the increases of brain weight, thickening of cerebral cortex and enlargement of hippocampus volume, etc. On cellular levels, EE could increase the proliferation of neural progenitor cells, production of newly generated neurons, the dendritic branches and tree density and the number of neuronal synapsis. These changes are most obvious in hippocampus and cerebral cortex. EE also could change the morphology of glia cells (including astrocyte and oligodendroglia cell) and promote the glial cell proliferation in the brain and cerebellum. EE could also induce various neural active substance changes, BDNF, VEGF, NGF and so on. These growth factors play important role on neurogenesis and neural network, which helps neuron development, differentiation and survival. In these ways, EE could improve the learning ability and cognition.

Several questions remain unclear. Classic water maze was usually applied in mice around 8 months old. However, most EE studies used transgenic AD mice of relative young age, about 2–3 month-old. Thus, the effect of EE in elder mice was still unclarified. Maybe older mice should be used on EE in the following days. Besides, few study concerns how EE influence neuronal death and related mechanism for its non-ignorable role in neural circuit. A more difficult challenge is linking animal model data with clinical studies. Finally, the questions we raised in Section Why we turn to bench for more help? were only partly answered in animal studies. We could conclude that the pathological changes in AD model could be modified by cognitive interventions. The best duration and long-term effect of these intervention still remain unclear. An overall systematic explanation for the internal mechanism of EE should be given in further studies.

## Abbreviations

AD: Alzheimer’s disease
APP: Myloid precursor protein
BDNF: Brain-derived neurotrophic factor
BOLD: Blood oxygenation level dependent contrast
ChEIs: Cholinesterase inhibitors
CNS: Central nervous system
CREB: cAMP-response element binding protein
DG: Dentate gyrus
DTI: Diffusion tensor imaging
EE: Environmental enrichment
EEG: Electroencephalography
ERP: Event-related potentials
FDA: Food and drug administration
fMRI: Functional magnetic resonance imaging
IEG: Immediate-early gene
IGF: IGF nsulin-like growth factor
MCI: Mild cognitive impairment
MiR: microRNAs
NGF: Nerve growth factor
NMDAR: N-methyl-D-aspartic acid receptor
OB: Olfactory bulb
PRISMA: Preferred Reporting Items for Systematic Reviews and Meta-Analysis
PS: Presenilin
RNS: Reactive nitrogen species
ROS: Reactive oxygen species
SAMP8: Senescence-accelerated prone mice
SCF: Stem cell factor
SOD: Superoxide Dismutase
SVZ: Subventricular zone
TRPC1: Transient receptor potential-canonical 1
VEGF: Vascular endothelial growth factor
WT: Wild type

## References

[1] Brookmeyer R (2007). Forecasting the global burden of Alzheimer’s disease. Alzheimers Dement.

[2] Reitz C, Mayeux R (2014). Alzheimer disease: epidemiology, diagnostic criteria, risk factors and biomarkers. Biochem Pharmacol.

[3] Joyce AT (2007). Burden of illness among commercially insured patients with Alzheimer’s disease. Alzheimers Dement.

[4] Ding D (2014). Prevalence of mild cognitive impairment in an urban community in China: A cross-sectional analysis of the Shanghai Aging Study. Alzheimers Dement.

[5] Gauthier S (2006). Mild cognitive impairment. Lancet.

[6] Jia J (2014). The prevalence of mild cognitive impairment and its etiological subtypes in elderly Chinese. Alzheimers Dement.

[7] Ravaglia G (2008). Mild cognitive impairment: epidemiology and dementia risk in an elderly Italian population. J Am Geriatr Soc.

[8] Trinh NH (2003). Efficacy of cholinesterase inhibitors in the treatment of neuropsychiatric symptoms and functional impairment in Alzheimer disease: a meta-analysis. JAMA.

[9] Reisberg B (2003). Memantine in moderate-to-severe Alzheimer’s disease. N Engl J Med.

[10] Raina P (2008). Effectiveness of cholinesterase inhibitors and memantine for treating dementia: evidence review for a clinical practice guideline. Ann Intern Med.

[11] Courtney C (2004). Long-term donepezil treatment in 565 patients with Alzheimer’s disease (AD2000): randomised double-blind trial. Lancet.

[12] Russ TC, Morling JR (2012). Cholinesterase inhibitors for mild cognitive impairment. Cochrane Database Syst Rev.

[13] Schneider LS (2011). Treatment with cholinesterase inhibitors and memantine of patients in the Alzheimer’s Disease Neuroimaging Initiative. Arch Neurol.

[14] Olazaran J (2010). Nonpharmacological therapies in Alzheimer’s disease: a systematic review of efficacy. Dement Geriatr Cogn Disord.

[15] Buschert V, Bokde AL, Hampel H (2010). Cognitive intervention in Alzheimer disease. Nat Rev Neurol.

[16] Reijnders J, van Heugten C, van Boxtel M (2013). Cognitive interventions in healthy older adults and people with mild cognitive impairment: a systematic review. Ageing Res Rev.

[17] Jean L (2010). Cognitive intervention programs for individuals with mild cognitive impairment: systematic review of the literature. Am J Geriatr Psychiatry.

[18] Simon SS, Yokomizo JE, Bottino CM (2012). Cognitive intervention in amnestic Mild Cognitive Impairment: a systematic review. Neurosci Biobehav Rev.

[19] Lampit A, Hallock H, Valenzuela M (2014). Computerized cognitive training in cognitively healthy older adults: a systematic review and meta-analysis of effect modifiers. PLoS Med.

[20] Clare L (2003). Cognitive rehabilitation and cognitive training for early-stage Alzheimer’s disease and vascular dementia. Cochrane Database Syst Rev.

[21] Grandmaison E, Simard M (2003). A critical review of memory stimulation programs in Alzheimer’s disease. J Neuropsychiatry Clin Neurosci.

[22] Baltes, P. B. and S. L. Willis. Plasticity and Enhancement of Intellectual Functioning in Old Age. Aging and Cognitive Processes. F. I. M. Craik and S. Trehub. Boston, MA: Springer US; 1982: 353-89.

[23] Yesavage JA. Nonpharmacologic treatments for memory losses with normal aging. Am J Psychiatry. 1985; 142(5):600-5. 10.1176/ajp.142.5.600 3885762

[24] Greenberg C, Powers SM (1987). Memory improvement among adult learners. Educ Gerontol.

[25] Rebok GW, Balcerak LJ (1989). Memory self-efficacy and performance differences in young and old adults: The effect of mnemonic training. Dev Psychol.

[26] Rebok GW, Rasmusson D, Brandt J (1996). Prospects for computerized memory training in normal elderly: Effects of practice on explicit and implicit memory tasks. Appl Cogn Psychol.

[27] Rasmusson DX (1999). Effects of three types of memory training in normal elderly. Aging Neuropsychol Cogn.

[28] Fairchild JK, Scogin FR (2010). Training to Enhance Adult Memory (TEAM): an investigation of the effectiveness of a memory training program with older adults. Aging Ment Health.

[29] McDougall GJ (2010). The SeniorWISE study: improving everyday memory in older adults. Arch Psychiatr Nurs.

[30] Buschkuehl M (2008). Impact of working memory training on memory performance in old-old adults. Psychol Aging.

[31] Melby-Lervag M, Hulme C. There is no convincing evidence that working memory training is effective: A reply to Au et al. (2014) and Karbach and Verhaeghen (2014). Psychon Bull Rev. 2016; 23(1):324-30. 10.3758/s13423-015-0862-z 26082279

[32] Karbach J, Verhaeghen P (2014). Making working memory work: a meta-analysis of executive-control and working memory training in older adults. Psychol Sci.

[33] Li SC (2008). Working memory plasticity in old age: practice gain, transfer, and maintenance. Psychol Aging.

[34] Berry AS (2010). The influence of perceptual training on working memory in older adults. PLoS ONE.

[35] Borella E (2010). Working memory training in older adults: evidence of transfer and maintenance effects. Psychol Aging.

[36] Jobe JB (2001). ACTIVE: a cognitive intervention trial to promote independence in older adults. Control Clin Trials.

[37] Ball K (2002). Effects of cognitive training interventions with older adults: a randomized controlled trial. JAMA.

[38] Rebok GW (2014). Ten-year effects of the advanced cognitive training for independent and vital elderly cognitive training trial on cognition and everyday functioning in older adults. J Am Geriatr Soc.

[39] Ross LA (2016). The Transfer of Cognitive Speed of Processing Training to Older Adults’ Driving Mobility Across 5 Years. J Gerontol B Psychol Sci Soc Sci.

[40] Wolinsky FD (2016). Effects of cognitive speed of processing training on a composite neuropsychological outcome: results at one-year from the IHAMS randomized controlled trial. Int Psychogeriatr.

[41] Buiza C (2008). A randomized, two-year study of the efficacy of cognitive intervention on elderly people: the Donostia Longitudinal Study. Int J Geriatr Psychiatry.

[42] Smith GE (2009). A cognitive training program based on principles of brain plasticity: results from the Improvement in Memory with Plasticity-based Adaptive Cognitive Training (IMPACT) study. J Am Geriatr Soc.

[43] Diamond K (2015). Randomized controlled trial of a healthy brain ageing cognitive training program: effects on memory, mood, and sleep. J Alzheimers Dis.

[44] Carlson MC (2008). Exploring the effects of an “everyday” activity program on executive function and memory in older adults: Experience Corps. Gerontologist.

[45] Tranter LJ, Koutstaal W (2008). Age and flexible thinking: an experimental demonstration of the beneficial effects of increased cognitively stimulating activity on fluid intelligence in healthy older adults. Neuropsychol Dev Cogn B Aging Neuropsychol Cogn.

[46] Wagner S (2008). Does a cognitive-training programme improve the performance of middle-aged employees undergoing in-patient psychosomatic treatment?. Disabil Rehabil.

[47] Ngandu T, et al. A 2 year multidomain intervention of diet, exercise, cognitive training, and vascular risk monitoring versus control to prevent cognitive decline in at-risk elderly people (FINGER): a randomised controlled trial. Lancet. 2015;385(9984):2255-63. 10.1016/S0140-6736(15)60461-5 25771249

[48] Rozzini L (2007). Efficacy of cognitive rehabilitation in patients with mild cognitive impairment treated with cholinesterase inhibitors. Int J Geriatr Psychiatry.

[49] Talassi E (2007). Effectiveness of a cognitive rehabilitation program in mild dementia (MD) and mild cognitive impairment (MCI): a case control study. Arch Gerontol Geriatr.

[50] Wenisch E (2007). Cognitive stimulation intervention for elders with mild cognitive impairment compared with normal aged subjects: preliminary results. Aging Clin Exp Res.

[51] Kurz A (2009). Cognitive rehabilitation in patients with mild cognitive impairment.

[52] Greenaway MC (2008). A behavioral rehabilitation intervention for amnestic mild cognitive impairment. Am J Alzheimers Dis Other Demen.

[53] Barnes DE (2009). Computer-based cognitive training for mild cognitive impairment: results from a pilot randomized, controlled trial. Alzheimer Dis Assoc Disord.

[54] Bottiroli S, Cavallini E (2009). Can computer familiarity regulate the benefits of computer-based memory training in normal aging? A study with an Italian sample of older adults. Neuropsychol Dev Cogn B Aging Neuropsychol Cogn.

[55] Rapp S, Brenes G, Marsh AP (2002). Memory enhancement training for older adults with mild cognitive impairment: a preliminary study. Aging Ment Health.

[56] Cappelletti M (2013). Transfer of Cognitive Training across Magnitude Dimensions Achieved with Concurrent Brain Stimulation of the Parietal Lobe. J Neurosci.

[57] Belleville S (2006). Improvement of episodic memory in persons with mild cognitive impairment and healthy older adults: evidence from a cognitive intervention program. Dement Geriatr Cogn Disord.

[58] Hampstead BM (2008). Explicit memory training leads to improved memory for face-name pairs in patients with mild cognitive impairment: results of a pilot investigation. J Int Neuropsychol Soc.

[59] Vranic A, et al. The efficacy of a multifactorial memory training in older adults living in residential care settings. Int Psychogeriatr. 2013;25(11):1885-97. 10.1017/S1041610213001154 23899952

[60] Lovden M (2010). A theoretical framework for the study of adult cognitive plasticity. Psychol Bull.

[61] Londos E (2008). Effects of a goal-oriented rehabilitation program in mild cognitive impairment: a pilot study. Am J Alzheimers Dis Other Demen.

[62] Troyer AK (2008). Changing everyday memory behaviour in amnestic mild cognitive impairment: a randomised controlled trial. Neuropsychol Rehabil.

[63] Quayhagen MP (2000). Coping with dementia: evaluation of four nonpharmacologic interventions. Int Psychogeriatr.

[64] Zarit SH, Zarit JM, Reever KE (1982). Memory training for severe memory loss: effects on senile dementia patients and their families. Gerontologist.

[65] Backman L (1991). The generalizability of training gains in dementia: effects of an imagery-based mnemonic on face-name retention duration. Psychol Aging.

[66] Davis RN, Massman PJ, Doody RS (2001). Cognitive intervention in Alzheimer disease: a randomized placebo-controlled study. Alzheimer Dis Assoc Disord.

[67] Clare L (2000). Intervening with everyday memory problems in dementia of Alzheimer type: an errorless learning approach. J Clin Exp Neuropsychol.

[68] Haslam C, Hodder KI, Yates PJ (2011). Errorless learning and spaced retrieval: how do these methods fare in healthy and clinical populations?. J Clin Exp Neuropsychol.

[69] Landauer TK, Bjork RA (1978). Optimum rehearsal patterns and name learning. Pract Asp Mem.

[70] Hawley KS (2008). A comparison of adjusted spaced retrieval versus a uniform expanded retrieval schedule for learning a name-face association in older adults with probable Alzheimer’s disease. J Clin Exp Neuropsychol.

[71] Small JA (2012). A new frontier in spaced retrieval memory training for persons with Alzheimer’s disease. Neuropsychol Rehabil.

[72] McKitrick LA, Camp CJ, Black FW (1992). Prospective memory intervention in Alzheimer’s disease. J Gerontol.

[73] Kixmiller JS (2002). Evaluation of prospective memory training for individuals with mild Alzheimer’s disease. Brain Cogn.

[74] Hampstead BM (2011). Activation and effective connectivity changes following explicit-memory training for face-name pairs in patients with mild cognitive impairment: a pilot study. Neurorehabil Neural Repair.

[75] Rosen AC (2011). Cognitive training changes hippocampal function in mild cognitive impairment: a pilot study. J Alzheimers Dis.

[76] Belleville S (2011). Training-related brain plasticity in subjects at risk of developing Alzheimer’s disease. Brain.

[77] Chapman SB. et al. Neural Mechanisms of Brain Plasticity with Complex Cognitive Training in Healthy Seniors. Cereb Cortex. 2013;25(2):396-405. 10.1093/cercor/bht234 PMC4351428 23985135

[78] Sagi Y (2012). Learning in the fast lane: new insights into neuroplasticity. Neuron.

[79] Thomas C, Baker CI (2013). Teaching an adult brain new tricks: a critical review of evidence for training-dependent structural plasticity in humans. Neuroimage.

[80] Strenziok M. et al. Neurocognitive enhancement in older adults: Comparison of three cognitive training tasks to test a hypothesis of training transfer in brain connectivity. Neuroimage. 2014;15;85Pt 3: 1027-39. 10.1016/j.neuroimage.2013.07.069 23933474

[81] Sauseng P (2007). Dissociation of sustained attention from central executive functions: local activity and interregional connectivity in the theta range. Eur J Neurosci.

[82] Onton J, Delorme A, Makeig S (2005). Frontal midline EEG dynamics during working memory. Neuroimage.

[83] Anguera JA (2013). Video game training enhances cognitive control in older adults. Nature.

[84] Albouy G (2012). Neural correlates of performance variability during motor sequence acquisition. Neuroimage.

[85] O’Brien JL. et al. Cognitive training and selective attention in the aging brain: An electrophysiological study. Clin Neurophysiol. 2013;124(11):2198-208. 10.1016/j.clinph.2013.05.012 23770088

[86] Li BY (2015). Mental Training for Cognitive Improvement in Elderly People: What have We Learned from Clinical and Neurophysiologic Studies?. Curr Alzheimer Res.

[87] Martinez-Moreno M., et al. Comparison of neuropsychological and functional outcomes in Alzheimer’s disease patients with good or bad response to a cognitive stimulation treatment: a retrospective analysis. Int Psychogeriatr. 2016;28(11):1–13. 10.1017/S104161021600123X 27503001

[88] Jean L (2007). Towards a cognitive stimulation program using an errorless learning paradigm in amnestic mild cognitive impairment. Neuropsychiatr Dis Treat.

[89] Jean L (2010). Efficacy of a cognitive training programme for mild cognitive impairment: results of a randomised controlled study. Neuropsychol Rehabil.

[90] Unverzagt FW (2007). Effect of memory impairment on training outcomes in ACTIVE. J Int Neuropsychol Soc.

[91] Kinsella GJ (2009). Early intervention for mild cognitive impairment: a randomised controlled trial. J Neurol Neurosurg Psychiatry.

[92] He Y (2007). Regional coherence changes in the early stages of Alzheimer’s disease: a combined structural and resting-state functional MRI study. Neuroimage.

[93] Cha J (2015). Assessment of Functional Characteristics of Amnestic Mild Cognitive Impairment and Alzheimer’s Disease Using Various Methods of Resting-State FMRI Analysis. Biomed Res Int.

[94] Chen Y (2016). Functional Activity and Connectivity Differences of Five Resting-State Networks in Patients with Alzheimer’s Disease or Mild Cognitive Impairment. Curr Alzheimer Res.

[95] Whitwell JL (2015). Working memory and language network dysfunctions in logopenic aphasia: a task-free fMRI comparison with Alzheimer’s dementia. Neurobiol Aging.

[96] Eklund A, Nichols TE, Knutsson H (2016). Cluster failure: Why fMRI inferences for spatial extent have inflated false-positive rates. Proc Natl Acad Sci U S A.

[97] Toth LA (2011). Environmental enrichment of laboratory rodents: the answer depends on the question. Comp Med.

[98] Hebb DO (1947). The effects of early experience on problem solving at maturity. Am Psychol.

[99] Krech D, Rosenzweig MR, Bennett EL (1960). Effects of environmental complexity and training on brain chemistry. J Comp Physiol Psychol.

[100] Rosenzweig MR (1962). Effects of environmental complexity and training on brain chemistry and anatomy: a replication and extension. J Comp Physiol Psychol.

[101] Diamond MC, Krech D, Rosenzweig MR (1964). The Effects of an Enriched Environment on the Histology of the Rat Cerebral Cortex. J Comp Neurol.

[102] Bennett EL, Rosenzweig MR, Diamond MC (1969). Rat brain: effects of environmental enrichment on wet and dry weights. Science.

[103] Altman J, Das GD (1964). Autoradiographic Examination of the Effects of Enriched Environment on the Rate of Glial Multiplication in the Adult Rat Brain. Nature.

[104] Pham TM (1999). Changes in brain nerve growth factor levels and nerve growth factor receptors in rats exposed to environmental enrichment for one year. Neuroscience.

[105] Ickes BR (2000). Long-term environmental enrichment leads to regional increases in neurotrophin levels in rat brain. Exp Neurol.

[106] Rampon C (2000). Effects of environmental enrichment on gene expression in the brain. Proc Natl Acad Sci U S A.

[107] Passineau MJ, Green EJ, Dietrich WD (2001). Therapeutic effects of environmental enrichment on cognitive function and tissue integrity following severe traumatic brain injury in rats. Exp Neurol.

[108] Xerri C, Zennou-Azougui Y, Coq JO (2003). Neuroprotective effects on somatotopic maps resulting from piracetam treatment and environmental enrichment after focal cortical injury. ILAR J.

[109] Guilarte TR (2003). Environmental enrichment reverses cognitive and molecular deficits induced by developmental lead exposure. Ann Neurol.

[110] Dahlqvist P (2004). Environmental enrichment reverses learning impairment in the Morris water maze after focal cerebral ischemia in rats. Eur J Neurosci.

[111] Morley-Fletcher S (2003). Environmental enrichment during adolescence reverses the effects of prenatal stress on play behaviour and HPA axis reactivity in rats. Eur J Neurosci.

[112] Bartoletti A (2004). Environmental enrichment prevents effects of dark-rearing in the rat visual cortex. Nat Neurosci.

[113] Frick KM, Fernandez SM (2003). Enrichment enhances spatial memory and increases synaptophysin levels in aged female mice. Neurobiol Aging.

[114] Duffy SN (2001). Environmental enrichment modifies the PKA-dependence of hippocampal LTP and improves hippocampus-dependent memory. Learn Mem.

[115] Leggio MG (2005). Environmental enrichment promotes improved spatial abilities and enhanced dendritic growth in the rat. Behav Brain Res.

[116] Hicks RR (2002). Environmental enrichment attenuates cognitive deficits, but does not alter neurotrophin gene expression in the hippocampus following lateral fluid percussion brain injury. Neuroscience.

[117] Dobrossy MD, Dunnett SB (2004). Environmental enrichment affects striatal graft morphology and functional recovery. Eur J Neurosci.

[118] Arendash GW (2004). Environmental enrichment improves cognition in aged Alzheimer’s transgenic mice despite stable beta-amyloid deposition. Neuroreport.

[119] Jankowsky JL (2005). Environmental enrichment mitigates cognitive deficits in a mouse model of Alzheimer’s disease. J Neurosci.

[120] Lazarov O (2005). Environmental enrichment reduces Abeta levels and amyloid deposition in transgenic mice. Cell.

[121] De Rosa R (2005). Intranasal administration of nerve growth factor (NGF) rescues recognition memory deficits in AD11 anti-NGF transgenic mice. Proc Natl Acad Sci U S A.

[122] Capsoni S (2000). Alzheimer-like neurodegeneration in aged antinerve growth factor transgenic mice. Proc Natl Acad Sci U S A.

[123] Berardi N (2007). Environmental enrichment delays the onset of memory deficits and reduces neuropathological hallmarks in a mouse model of Alzheimer-like neurodegeneration. J Alzheimers Dis.

[124] Gortz N (2008). Effects of environmental enrichment on exploration, anxiety, and memory in female TgCRND8 Alzheimer mice. Behav Brain Res.

[125] Herring A (2010). Reduction of cerebral oxidative stress following environmental enrichment in mice with Alzheimer-like pathology. Brain Pathol.

[126] Jeong YH (2011). Environmental enrichment compensates for the effects of stress on disease progression in Tg2576 mice, an Alzheimer’s disease model. J Neurochem.

[127] Beauquis J (2013). Environmental enrichment prevents astroglial pathological changes in the hippocampus of APP transgenic mice, model of Alzheimer’s disease. Exp Neurol.

[128] Polito L (2014). Environmental enrichment lessens cognitive decline in APP23 mice without affecting brain sirtuin expression. J Alzheimers Dis.

[129] Lahiani-Cohen I (2011). Moderate environmental enrichment mitigates tauopathy in a neurofibrillary tangle mouse model. J Neuropathol Exp Neurol.

[130] Grinan-Ferre C (2016). Environmental Enrichment Improves Behavior, Cognition, and Brain Functional Markers in Young Senescence-Accelerated Prone Mice (SAMP8). Mol Neurobiol.

[131] Altman J, Das GD (1966). Autoradiographic and histological studies of postnatal neurogenesis. I. A longitudinal investigation of the kinetics, migration and transformation of cells incorporating tritiated thymidine in neonate rats, with special reference to postnatal neurogenesis in some brain regions. J Comp Neurol.

[132] Lois C, Alvarez-Buylla A (1994). Long-distance neuronal migration in the adult mammalian brain. Science.

[133] Kornack DR, Rakic P (2001). The generation, migration, and differentiation of olfactory neurons in the adult primate brain. Proc Natl Acad Sci U S A.

[134] Mouret A (2009). Turnover of newborn olfactory bulb neurons optimizes olfaction. J Neurosci.

[135] Kageyama R, Imayoshi I, Sakamoto M (2012). The role of neurogenesis in olfaction-dependent behaviors. Behav Brain Res.

[136] Sakamoto M (2011). Continuous neurogenesis in the adult forebrain is required for innate olfactory responses. Proc Natl Acad Sci U S A.

[137] Squire LR (1992). Memory and the hippocampus: a synthesis from findings with rats, monkeys, and humans. Psychol Rev.

[138] Sahay A (2011). Increasing adult hippocampal neurogenesis is sufficient to improve pattern separation. Nature.

[139] Stone SS (2011). Stimulation of entorhinal cortex promotes adult neurogenesis and facilitates spatial memory. J Neurosci.

[140] Kuhn HG, Dickinson-Anson H, Gage FH (1996). Neurogenesis in the dentate gyrus of the adult rat: age-related decrease of neuronal progenitor proliferation. J Neurosci.

[141] Kempermann G, Kuhn HG, Gage FH (1997). More hippocampal neurons in adult mice living in an enriched environment. Nature.

[142] Lazarov O, Marr RA (2010). Neurogenesis and Alzheimer’s disease: at the crossroads. Exp Neurol.

[143] Lopez-Toledano MA (2010). Adult neurogenesis: a potential tool for early diagnosis in Alzheimer’s disease?. J Alzheimers Dis.

[144] Brinton RD, Wang JM (2006). Therapeutic potential of neurogenesis for prevention and recovery from Alzheimer’s disease: allopregnanolone as a proof of concept neurogenic agent. Curr Alzheimer Res.

[145] Donovan MH (2006). Decreased adult hippocampal neurogenesis in the PDAPP mouse model of Alzheimer’s disease. J Comp Neurol.

[146] Rogers J (2016). Dissociating the therapeutic effects of environmental enrichment and exercise in a mouse model of anxiety with cognitive impairment. Transl Psychiatry.

[147] Leger M (2015). Environmental Enrichment Duration Differentially Affects Behavior and Neuroplasticity in Adult Mice. Cereb Cortex.

[148] Du LL, et al. Transient Receptor Potential-canonical 1 is Essential for Environmental Enrichment-Induced Cognitive Enhancement and Neurogenesis. Mol Neurobiol. 2017;54(3):1992-2002. 10.1007/s12035-016-9758-9 26910815

[149] Kempermann G, Gast D, Gage FH (2002). Neuroplasticity in old age: sustained fivefold induction of hippocampal neurogenesis by long-term environmental enrichment. Ann Neurol.

[150] Mirochnic S (2009). Age effects on the regulation of adult hippocampal neurogenesis by physical activity and environmental enrichment in the APP23 mouse model of Alzheimer disease. Hippocampus.

[151] Wolf SA (2006). Cognitive and physical activity differently modulate disease progression in the amyloid precursor protein (APP)-23 model of Alzheimer’s disease. Biol Psychiatry.

[152] Costa DA (2007). Enrichment improves cognition in AD mice by amyloid-related and unrelated mechanisms. Neurobiol Aging.

[153] Dong S (2007). Environment enrichment rescues the neurodegenerative phenotypes in presenilins-deficient mice. Eur J Neurosci.

[154] Valero J (2011). Short-term environmental enrichment rescues adult neurogenesis and memory deficits in APP(Sw, Ind) transgenic mice. PLoS ONE.

[155] Hu YS (2010). Complex environment experience rescues impaired neurogenesis, enhances synaptic plasticity, and attenuates neuropathology in familial Alzheimer’s disease-linked APPswe/PS1DeltaE9 mice. FASEB J.

[156] Brown J (2003). Enriched environment and physical activity stimulate hippocampal but not olfactory bulb neurogenesis. Eur J Neurosci.

[157] Veyrac A (2009). Novelty determines the effects of olfactory enrichment on memory and neurogenesis through noradrenergic mechanisms. Neuropsychopharmacology.

[158] Shapiro LA (2007). Olfactory enrichment enhances the survival of newly born cortical neurons in adult mice. Neuroreport.

[159] Matsumori Y (2006). Enriched environment and spatial learning enhance hippocampal neurogenesis and salvages ischemic penumbra after focal cerebral ischemia. Neurobiol Dis.

[160] Ehninger D, Kempermann G (2003). Regional effects of wheel running and environmental enrichment on cell genesis and microglia proliferation in the adult murine neocortex. Cereb Cortex.

[161] Kronenberg G (2007). Local origin and activity-dependent generation of nestin-expressing protoplasmic astrocytes in CA1. Brain Struct Funct.

[162] Viola GG (2009). Morphological changes in hippocampal astrocytes induced by environmental enrichment in mice. Brain Res.

[163] Williamson LL, Chao A, Bilbo SD (2012). Environmental enrichment alters glial antigen expression and neuroimmune function in the adult rat hippocampus. Brain Behav Immun.

[164] Olsson T (1995). Transcription factor AP-2 gene expression in adult rat hippocampal regions: effects of environmental manipulations. Neurosci Lett.

[165] Gagne J (1998). AMPA receptor properties in adult rat hippocampus following environmental enrichment. Brain Res.

[166] Rasmuson S (1998). Environmental enrichment selectively increases 5-HT1A receptor mRNA expression and binding in the rat hippocampus. Brain Res Mol Brain Res.

[167] Andin J (2007). Influence of environmental enrichment on steady-state mRNA levels for EAAC1, AMPA1 and NMDA2A receptor subunits in rat hippocampus. Brain Res.

[168] Munetsuna E (2011). Environmental enrichment alters gene expression of steroidogenic enzymes in the rat hippocampus. Gen Comp Endocrinol.

[169] Keyvani K (2004). Gene expression profiling in the intact and injured brain following environmental enrichment. J Neuropathol Exp Neurol.

[170] Toscano CD, McGlothan JL, Guilarte TR (2006). Experience-dependent regulation of zif268 gene expression and spatial learning. Exp Neurol.

[171] Li C (2007). Effects of enriched environment on gene expression and signal pathways in cortex of hippocampal CA1 specific NMDAR1 knockout mice. Brain Res Bull.

[172] Huang FL, Huang KP, Boucheron C (2007). Long-term enrichment enhances the cognitive behavior of the aging neurogranin null mice without affecting their hippocampal LTP. Learn Mem.

[173] Patel CJ, Butte AJ (2010). Predicting environmental chemical factors associated with disease-related gene expression data. BMC Med Genomics.

[174] Ragu Varman D, Marimuthu G, Rajan KE (2013). Environmental enrichment upregulates micro-RNA-183 and alters acetylcholinesterase splice variants to reduce anxiety-like behavior in the little Indian field mouse (Mus booduga). J Neurosci Res.

[175] Durairaj RV, Koilmani ER (2014). Environmental enrichment modulates glucocorticoid receptor expression and reduces anxiety in Indian field male mouse Mus booduga through up-regulation of microRNA-124a. Gen Comp Endocrinol.

[176] Barak B (2013). Opposing actions of environmental enrichment and Alzheimer’s disease on the expression of hippocampal microRNAs in mouse models. Transl Psychiatry.

[177] Miranda S (2000). The role of oxidative stress in the toxicity induced by amyloid beta-peptide in Alzheimer’s disease. Prog Neurobiol.

[178] Fernandez CI (2004). Environmental enrichment-behavior-oxidative stress interactions in the aged rat: issues for a therapeutic approach in human aging. Ann N Y Acad Sci.

[179] Arranz L (2010). Environmental enrichment improves age-related immune system impairment: long-term exposure since adulthood increases life span in mice. Rejuvenation Res.

[180] Marmol F (2015). Anti-oxidative effects produced by environmental enrichment in the hippocampus and cerebral cortex of male and female rats. Brain Res.

[181] Herring A (2011). Preventive and therapeutic types of environmental enrichment counteract beta amyloid pathology by different molecular mechanisms. Neurobiol Dis.

[182] Goshen I (2009). Environmental enrichment restores memory functioning in mice with impaired IL-1 signaling via reinstatement of long-term potentiation and spine size enlargement. J Neurosci.

[183] Sun H (2010). Environmental enrichment influences BDNF and NR1 levels in the hippocampus and restores cognitive impairment in chronic cerebral hypoperfused rats. Curr Neurovasc Res.

[184] Rossi C (2006). Brain-derived neurotrophic factor (BDNF) is required for the enhancement of hippocampal neurogenesis following environmental enrichment. Eur J Neurosci.

[185] Bekinschtein P (2011). Effects of environmental enrichment and voluntary exercise on neurogenesis, learning and memory, and pattern separation: BDNF as a critical variable?. Semin Cell Dev Biol.

[186] Novkovic T, Mittmann T, Manahan-Vaughan D (2015). BDNF contributes to the facilitation of hippocampal synaptic plasticity and learning enabled by environmental enrichment. Hippocampus.

[187] Hu YS (2013). Molecular mechanisms of environmental enrichment: impairments in Akt/GSK3beta, neurotrophin-3 and CREB signaling. PLoS ONE.

[188] Pham TM (1999). Effects of environmental enrichment on cognitive function and hippocampal NGF in the non-handled rats. Behav Brain Res.

[189] Angelucci F (2009). Increased concentrations of nerve growth factor and brain-derived neurotrophic factor in the rat cerebellum after exposure to environmental enrichment. Cerebellum.

[190] Torasdotter M (1998). Environmental enrichment results in higher levels of nerve growth factor mRNA in the rat visual cortex and hippocampus. Behav Brain Res.

[191] Birch AM, McGarry NB, Kelly AM (2013). Short-term environmental enrichment, in the absence of exercise, improves memory, and increases NGF concentration, early neuronal survival, and synaptogenesis in the dentate gyrus in a time-dependent manner. Hippocampus.

[192] Kelly ME (2014). The impact of cognitive training and mental stimulation on cognitive and everyday functioning of healthy older adults: a systematic review and meta-analysis. Ageing Res Rev.

[193] Curlik DM, Shors TJ (2011). Learning Increases the Survival of Newborn Neurons Provided That Learning Is Difficult to Achieve and Successful. J Cogn Neurosci.

[194] Dahlin E (2008). Transfer of learning after updating training mediated by the striatum. Science.

[195] Mahncke HW, Bronstone A, Merzenich MM (2006). Brain plasticity and functional losses in the aged: scientific bases for a novel intervention. Prog Brain Res.

[196] Logsdon RG (2002). Assessing quality of life in older adults with cognitive impairment. Psychosom Med.

[197] Alexopoulos GS (1988). Cornell Scale for Depression in Dementia. Biol Psychiatry.

[198] Thivierge S, Jean L, Simard M. A Randomized Cross-over Controlled Study on Cognitive Rehabilitation of Instrumental Activities of Daily Living in Alzheimer Disease. Am J Geriatr Psychiatry. 2014;22(11):1188-99. 10.1016/j.jagp.2013.03.008 23871120

[199] Mayeux R (2003). Epidemiology of neurodegeneration. Annu Rev Neurosci.

[200] Toril P, Reales JM, Ballesteros S (2014). Video game training enhances cognition of older adults: a meta-analytic study. Psychol Aging.

[201] Papp KV, Walsh SJ, Snyder PJ (2009). Immediate and delayed effects of cognitive interventions in healthy elderly: a review of current literature and future directions. Alzheimers Dement.

[202] Li H, Li J, Li N, Li B, Wang P, Zhou T (2011). Cognitive intervention for persons with mild cognitive impairment: A meta-analysis. Ageing Res Rev.

[203] Martin M, Clare L, Altgassen AM, Cameron MH, Zehnder F (2011). Cognition-based interventions for healthy older people and people with mild cognitive impairment. Cochrane Database Syst Rev.

[204] Bahar-Fuchs A, Clare L, Woods B (2013). Cognitive training and cognitive rehabilitation for mild to moderate Alzheimer’s disease and vascular dementia. Cochrane Database Syst Rev.

[205] Aguirre E, Woods RT, Spector A, Orrell M (2013). Cognitive stimulation for dementia: a systematic review of the evidence of effectiveness from randomised controlled trials. Ageing Res Rev.

[206] Sitzer DI, Twamley EW, Jeste DV (2006). Cognitive training in Alzheimer’s disease: a meta-analysis of the literature. Acta Psychiatr Scand.

[207] Woods B, Aguirre E, Spector AE, Orrell M (2012). Cognitive stimulation to improve cognitive functioning in people with dementia. Cochrane Database Syst Rev.

